# Draft Genome Sequences of Three Clinical Strains of Bacillus cereus Isolated from Human Patients in Japan

**DOI:** 10.1128/MRA.00415-19

**Published:** 2019-05-09

**Authors:** Akiko Okutani, Satoshi Inoue, Shigeru Morikawa

**Affiliations:** a Department of Veterinary Science, National Institute of Infectious Diseases, Tokyo, Japan; University of Rochester School of Medicine and Dentistry

## Abstract

Bacillus cereus is a common etiological agent of hospital-acquired infections. Here, we report the draft genome sequences of three clinical isolates of B. cereus (GTC2903, GTC2926, and ach14) isolated from three human patients in different hospitals and in different years in Japan.

## ANNOUNCEMENT

Bacillus cereus is a facultative, anaerobic, spore-forming, Gram-positive bacterium that is commonly present in soil (1), in the rhizosphere of plants (2, 3), and as part of animal intestinal microflora (4). B. cereus is an important foodborne pathogen and can cause two different types of gastrointestinal diseases, the emetic syndrome and the diarrheal syndrome (5). The bacterium is also an opportunistic pathogen causing various infections, including local infections of wounds, bacteremia, septicemia, respiratory infections, and central nervous system infections (6).

To understand the genetic background of clinical isolates of B. cereus, we sequenced the genomes of three B. cereus isolates derived from human patients with clinical symptoms. Strain ach14 was isolated in 2014 from the whole-blood culture of a patient with bacteremia, and the two GTC strains (GTC2903 and GTC2926) were isolated from different patients and were obtained from the National BioResource Project (NBRP) Center, Organization for Research and Community Development, Gifu University. Strain GTC2903 was isolated from a patient with an opportunistic infection in 1995, and strain GTC2926 was isolated from a patient blood culture in 2006. The identity of the three isolates was confirmed to be B. cereus by amplification and sequencing of the 16S rRNA genes.

Bacteria were grown in Luria-Bertani broth at 30°C for 18 h and pelleted. Genomic DNA was extracted from each pellet using a QIAamp DNA minikit (Qiagen, Hilden, Germany) and fragmented using an ultrasonicator to generate fragments between 300 bp and 400 bp. Libraries were prepared for MiSeq (Illumina, Inc.) sequencing according to the manufacturer’s instructions, using a NEBNext Ultra DNA library prep kit for Illumina (NEB Japan, Tokyo, Japan) with index primers from NEBNext Multiplex Oligos for Illumina (Set1 or Set2), and then sequenced using 300-bp paired-end sequencing with a MiSeq reagent kit v.3 (600 cycles). After filtering low-quality reads and quality trimming in CLC Genomics Workbench 11.0.1 (Qiagen) using default parameters, *de novo* assembly of high-quality paired-end reads was conducted using CLC Genomics Workbench 11.0.1 using standard settings. Genome annotation was performed using the DDBJ Fast Annotation and Submission Tool (DFAST) pipeline v.1.1.4 with default settings (7). Table 1 shows genome assembly and annotation metrics for the three isolates. The availability of these genomes will enable more comprehensive analyses of nosocomial infection by B. cereus.

**TABLE 1:** Genome assembly metrics and genetic features of three clinical B. cereus isolates

Isolate	No. of reads after trimming	G+C content (%)	No. of contigs	*N*_50_ (bp)	Estimated genome size (Mbp)	Total no. of genes	GenBank accession no.
GTC2903	1,330,006	35.2	66	197,233	6.04	6,278	BIYC00000000
GTC2926	4,141,174	35.3	62	161,410	5.42	5,565	BIYD00000000
ach14	2,387,596	35.0	43	292,928	5.32	5,375	BIYE00000000

### Data availability.

The genome sequences of the three Bacillus cereus strains from human patients have been deposited in DDBJ/GenBank under the accession numbers BIYC00000000 (GTC2903), BIYD00000000 (GTC2926), and BIYE00000000 (ach14). The raw sequence data were deposited in the DDBJ/Sequence Read Archive under accession numbers DRR168392, DRR168393, and DRR168394 for GTC2903, GTC2926, and ach14, respectively.
